# Physical Activity in Natural Environments Is Associated With Motivational Climate and the Prevention of Harmful Habits: Structural Equation Analysis

**DOI:** 10.3389/fpsyg.2019.01113

**Published:** 2019-05-29

**Authors:** Manuel Castro-Sánchez, Félix Zurita-Ortega, José Antonio Pérez-Turpin, Javier Cachón-Zagalaz, Cristian Cofre-Bolados, Concepción Suarez-Llorca, Ramón Chacón-Cuberos

**Affiliations:** ^1^Department of Didactics of Musical, Plastic and Body Expression, Faculty of Fine Arts, University of Granada, Granada, Spain; ^2^Department of Didactic General and Specific Training, University of Alicante, Alicante, Spain; ^3^Department of Didactics of Musical, Plastic and Body Expression, University of Jaén, Jaén, Spain; ^4^School of Physical Activity, Sports and Health Sciences, University of Santiago-Chile, Santiago, Chile; ^5^Escuela de Ciencias del Deporte, Facultad de Salud, Universidad Santo Tomás (UST), Santiago, Chile; ^6^Department of Evolutionary Psychology and Didactic, University of Alicante, Alicante, Spain; ^7^Departamento de Métodos de Investigación y Diagnóstico en Educación, Universidad de Granada, Granada, Spain

**Keywords:** motivation, physical activity, natural environment, alcohol, tobacco

## Abstract

**Background:**

Practicing physical activity in a natural environment has various benefits that make it an ideal setting to develop healthy behaviors and thereby diminish unhealthy habits. The objective of this study was to develop and verify an explicative model for motivational climate in sport, considering its potential influence on alcohol and tobacco consumption.

**Methods:**

The study included 2273 adolescents from Granada (Spain), analyzing motivational climate (PMCSQ-2), alcohol consumption (AUDIT) and tobacco consumption (FTND). Multi-group structural equation modeling was conducted, yielding an excellent fit (χ2 = 168.170; gl = 32; *p* = 0.00; CFI = 0.972; NFI = 0.966; IFI = 0.972; RMSEA = 0.045).

**Results:**

The main findings were: a negative relationship between task climate and alcohol consumption among students practicing physical activities in a natural environment; a positive relationship between ego climate and alcohol consumption among those practicing other types of physical activity; and a positive and direct relationship between alcohol and tobacco consumption, which was stronger among those who did not practice physical activity in a natural environment.

**Conclusion:**

We conclude that physical-sport activity practiced in nature is a key to acquiring healthy patterns characterized by intrinsic motivations in sport and lower consumption of harmful substances.

## Introduction

Adolescence represents a key stage in the development and acquisition of healthy and/or harmful habits that will endure throughout adulthood (Urrutia-Pereira et al., 2017). According to Lindgren et al. (2017) it is crucial to understand why tobacco and alcohol consumption begins during adolescence and to determine motivating factors, with the aim of avoiding the emergence of these behaviors and encouraging young people to acquire healthy life habits that persist during adulthood (Taylor et al., 2017).

Most of the research proposes the practice of healthy physical activity as a main alternative to these harmful habits (Barrett et al., 2017). In fact, it has been found a negative relationship between the levels of physical activity and the consumption of alcohol and tobacco, showing benefits at a physical, psychological and social level (Donoghue et al., 2017; Lewis et al., 2017). For these reasons, it is necessary to promote habits related to the practice of physical activity in children and adolescents towards in order to create healthy patterns that could be repeated in adulthood and avoid the development of maladaptative behaviors (Hatano et al., 2017; Montero-Duarte et al., 2019). There is a need to involve the peer group, educational institutions and family in the control of the multiple risk factors for this consumption in adolescents (Lebron et al., 2017).

Various researchers have proposed physical and sports activities as an alternative to these damaging behaviors and as a means of distancing young people from harmful substances (Cerkez et al., 2015). Ayán et al. (2017) reported that tobacco/alcohol consumption was inversely related to sports practice, which provides multiple psychological, physical and social benefits (Kose et al., 2015).

Physical activities in a natural environment have major hedonistic effects and involve interaction with nature in novel surrounds, while they are characterized by less strict rules in comparison to more competitive sports (Lawton et al., 2017). They may therefore be an ideal means for adolescents to acquire healthy habits, improving the physical condition of their whole body (Pretty et al., 2017; World Health Organization, 2017) and enhancing their psychological development in previously unknown settings that promote wellbeing and autonomy. Moreover, the socializing component of this type of activity fosters a feeling of belonging to the group and often requires collaboration to achieve its objectives (Sims-Gould et al., 2017).

Therefore, activities in natural environments appear to be an ideal way to get to know and enjoy the environment while performing motor activities that generate physical, cognitive and social benefits. This is because the natural environment has characteristics that make it conducive to fun, enhancing the sport’s hedonistic component (Lorenz et al., 2017). This type of physical activity has a strong motivational component, which is key to modifying the intensity and direction of behavior, because the activities carried out in the natural environment focus on fun and pleasure, eliminating the negative connotations of excessive competitiveness that is sometimes present in sports (Calogiuri and Elliott, 2017). Psychologists consider motivation as one of the most influential factors to explain human behaviors (Gutiérrez and López, 2012).

Various studies identified the benefits derived from the practice of physical activity in nature, classified as follows: a decrease in cardiovascular diseases, a decrease in the rates of overweight and obesity, preventive effect against type 2 diabetes, various psychosocial benefits such as reduced levels of anxiety, depression, stress and emotional distress (Casper and Pfahl, 2015; Cox et al., 2017; Gómez et al., 2018; Moreno et al., 2015).

The practice of physical activity carried out in the natural environment in a non-competitive way, in which the subject focuses on the achievement of intrinsic goals, orienting itself more towards intrinsic motivations, constitutes a factor of prevention against the consumption of alcohol and tobacco. Nevertheless, physical activity focused on the achievement of extrinsic goals, in which a motivational orientation towards the ego is adopted, may represent a risk for the consumption of these substances (Bengoechea et al., 2017).

The importance of motivation in people’s daily lives lies in their influence to perform certain behaviors or not, providing a theoretical basis to understand human behavior (Ryan and Deci, 2017). In the case of physical activity and sports, it will be valid to understand a multitude of psychological and behavioral factors related to their practice (Dweck, 1986). This study is focused on the motivational climate in sport, based on the Achievement Goal Theory (Nicholls, 1989) derived from the Self-determination Theory of Deci and Ryan (2008). According to this model, physical activities can be either ego-oriented, focused on demonstrating skills, competing with group members and defeating rivals, or task-oriented, centring on personal effort and self-improvement with greater self-determined motivation and enjoyment of the activity, enhancing adherence to physical activity practice (Newton et al., 2000; Palmer et al., 2017).

After reviewing the existing literature, the existence of an association between motivational climates and the development of healthy or maladaptive behaviors related to the consumption of harmful substances is observed. Therefore, it is important to analyze the possible relationship between the motivational climate in sports and the consumption of alcohol and tobacco, as well as to analyze the differences according to whether physical activity practice is performed in a standard way or in the natural environment because this can be constituted as a protective factor against this type of unhealthy habits. For these reasons, the objective of this study was to develop an explicative model for motivational climate in sport, considering its potential influence on alcohol and tobacco consumption. We performed multi-group structural equation modeling (SEM) as a function of the type of physical activity practiced by adolescents, comparing between activities in a natural environment and those in other settings.

## Materials and Methods

### Subjects and Design

A quantitative, descriptive and cross-sectional study was undertaken, followed by a relational study developed using Structural Equation Modeling to analyze the degree of dependence among the different study variables.

Participants were selected from among the 11030 secondary school students in the city of Granada in southern Spain (data provided by the Education Ministry of the Junta de Andalucía). A representative sample (0.02 error; 95.5% CI) was selected by stratification, proportionality and randomization techniques, considering sex (male-female) and cycle (first-second). The inclusion criteria were: a) that the adolescents attended Compulsory Secondary Education in the city of Granada; b) not have any type of pathology that prevents them from participating in the research: c) have the informed consent of the parents or legal guardians to participate in the study. The final sample included 2273 adolescents, aged between 13 and 17 years (*M* = 14.94 years; *SD* = 1.25). The study was approved by the Research Ethics Committee of the University of Granada (641/CEIH/2018) and followed the principles of the 1975 Helsinki Declaration. Informed consent was obtained from all participants and their guardians. The anonymity of the participants was preserved at all times.

### Measures

The perceived motivational climate in sport was evaluated with the 33-item Perceived Motivational Climate in Sport Questionnaire (PMCSQ-2), alongside the Spanish validation form González-Cutre et al. (2008). Participants responded to each item on a five-point scale from 1 (strongly disagree) to 5 (strongly agree). The questionnaire comprised two higher-order scales, each containing three subscales (task: cooperative learning [CL], effort/improvement [EI] and important role [IR]; ego: member rivalry [MR], unequal recognition [UR] and punishment for mistakes [PM]). Internal consistency of the data was assessed using Cronbach’s alpha and was acceptable for both perceived task-involved climate (TC) and perceived ego-involved climate (EC) subscales (α = 0.860 and α = 0.810, respectively).

Alcohol consumption was assessed using the Spanish adaptation by Rubio (1998) of the Alcohol Use Disorders Identification Test (AUDIT) (Saunders et al., 1993). AUDIT comprises ten items, the first eight are evaluated on a five-point Likert scale (0 = Never; 4 = Daily) and the last two items on a three-point Likert scale, yielding a score of 0, 2, or 4 points. The first three items of the AUDIT describe the frequency of consumption, the next three describe the level of dependence and the remaining four items are related to harmful consumption. Item scores are added together to produce an overall score for each dimension. Cronbach’s alpha for the data was α = 0.832.

Tobacco consumption was assessed using the Spanish adaptation by Villareal-González (2009) of the Fagerström Test for Nicotine Dependence (FTND) (Heatherton et al., 1991). This instrument evaluates the number or amount of cigarettes, impulse to smoke and nicotine dependency. It includes six questions, the first four are dichotomous (0 = No and 1 = Yes), and the other two follow a four-option Likert-type scale (0 = Never and 3 = Always). The sum of items ranges between 0 and 10, establishing four categories (0 = Non-smoker, 1–4 = Low dependency, 5–6 = Moderate dependency and >6 = High dependency). The reliability determined for this investigation was α = 0.956.

### Procedure

Permission was obtained from the Education Office of the Junta de Andalucía for the participation of secondary schools in the city of Granada, selected according to convenience sampling of the study categories. The management of each educational centre was informed about the nature of the research and the need for the collaboration of students. Authorisation forms were provided to obtain the informed consent of legal guardians.

Participants were guaranteed the anonymity of the information gathered, clarifying that it would only be used for scientific purposes. Interviewers were present during data gathering to solve any possible doubts, and no problems were reported. Teachers, counselors and other collaborators were thanked and promised a report on the data obtained, respecting confidentiality.

### Statistical Analysis

**FIGURE 1:**
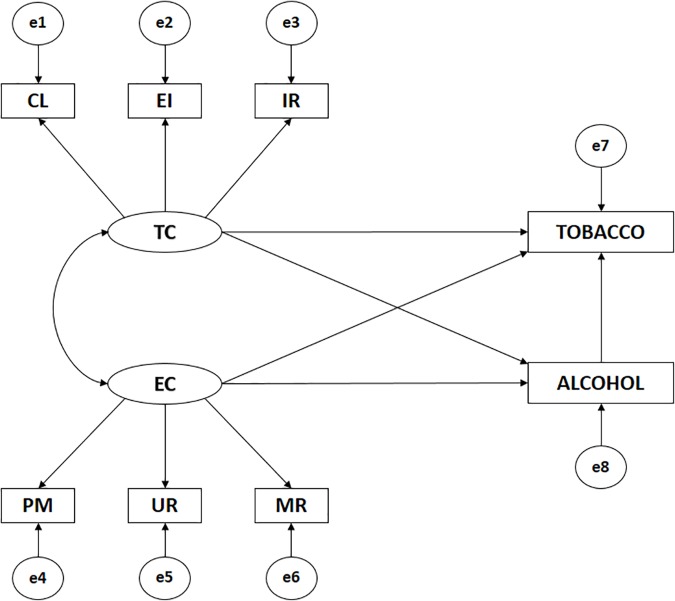
Model theories: Task climate, ego climate, alcohol consumption and tobacco consumption. TC, Climate-Task; CL, Climate-Task Cooperative Learning; EI, Climate-Task Effort/Improvement; IR, Climate-Task Important Role; EC, Climate-Ego; MR, Climate-ego Intra-team Member rivalry; UR, Climate-ego Unequal Recognition; PM, Climate-ego Punishment for Mistakes; TOBACCO, Tobacco Consumption; ALCOHOL, Alcohol Consumption.

SPSS^®^ version 22.0 (IBM Corp, Armonk, NY, United States) was used for data analyses. Descriptive analysis was performed, determining frequencies and mean values. Associative analysis was conducted using Pearson’s chi-square test. The significance level was set at 0.05. AMOS version 23.0 was used to create statistical models with instructive equations at the latent variable level. SEM was used to analyze the relationships between perceived motivational climate in sport, alcohol consumption and tobacco consumption (Figure 1). After developing the theoretical model, path analysis was conducted, considering the relationships of the matrix based on a multi-group analysis classifying participants in terms of the type of physical activity practiced as grouping variable (Marsh, 2007). Thus, two different structural models were configured to verify whether relationships among study variables varied as a function of the type of physical activity practiced: “physical activity in a natural environment” and “other type of physical activity.”

The SEM for these data is characterized by eight observed variables and two latent variables. This model provides causal explanations for the latent variables from the relationships observed between indicators. Squares represent the observed variables, circles the error terms, and ovals the latent variables. The exogenous latent variables, TC and EC, were each inferred using three indicators; for TC: CL, EI and IR; and for EC: MR, UR and PM. Alcohol consumption (ALCOHOL) and tobacco consumption (TOBACCO) were observed endogenous variables.

The bi-directional arrow (covariance) relates exogenous variables, while the unidirectional arrows are lines of influence between the latent and observable indicators, and are interpreted as multivariate regression coefficients. In addition, error prediction terms are associated with endogenous variables. The maximum likelihood (ML) method was used to estimate relationships between variables. We chose this method because it is consistent, unbiased and invariant to types of scale for variables with normal distribution.

Model fit was examined to verify the compatibility of the proposed model and the empirical information gathered. Goodness of fit was tested using a number of indexes briefly described here Chacón et al. (2018). Chi-square analysis was conducted when non-significant *p*-values indicated a good model fit. Comparative fit index (CFI), normalized fit index (NFI) and increase fit index (IFI) values >0.90 indicate acceptable model fit while values >0.95 indicate excellent model fit. Root mean square error of approximation (RMSEA) values <0.08 indicate acceptable model fit while values <0.05 indicate excellent model fit (Marsh, 2007).

## Results

Almost all model fit indexes indicated an excellent fit. A significant chi-square value was obtained (χ2 = 168.170; gl = 32; *p* < 0.001); however, given that this index has no upper limit and may also be sensitive to sample size, we also considered other standardized indexes less sensitive to sample size (Jöreskog, 1997). The NFI was 0.96, indicating an acceptable model fit. The CFI and the IFI yielded a value of 0.97 for both, indicating an excellent model fit. The RMSEA value was 0.45, indicating excellent fit (Browne and Cudeck, 1993). In summary, the data suggest that the model fits the empirical data well.

**FIGURE 2:**
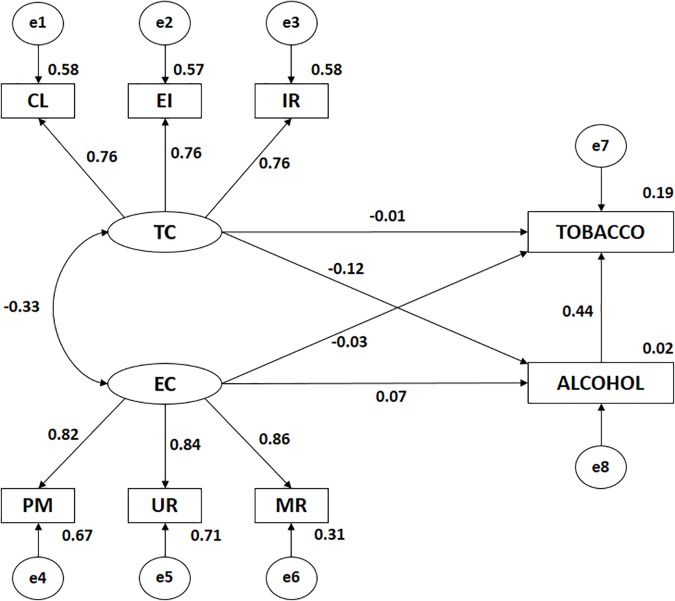
Structural equation model in those who practice physical activity in a natural environment. TC, Climate-Task; CL, Climate-Task Cooperative Learning; EI, Climate-Task Effort/Improvement; IR, Climate-Task Important Role; EC, Climate-Ego; MR, Climate-ego Intra-team Member rivalry; UR, Climate-ego Unequal Recognition; PM, Climate-ego Punishment for Mistakes; TOBACCO, Tobacco Consumption; ALCOHOL, Alcohol Consumption.

Figure 2 and Table 1 exhibit the estimated values of the structural model parameters for students who practiced physical activity in a natural environment. They should present adequate magnitude, and the effects should significantly differ from zero. Likewise, improper estimations should not be obtained, such as negative variances. Statistically significant positive and direct relationships (*p* < 0.005) were observed among all categories of motivational climate and its dimensions. There was a significant (*p* < 0.005) negative indirect relationship between TC and EC (*r* = -0.331).

**Table 1:** Weights and standardized regression weights of those who practice physical activity in a natural environment.

Relationship between variables	RW				SRW
	Estimations	SE	CR	*P*	Estimations
ALCOHOL ← EC	0.736	0.334	2.206	0.027	0.069
ALCOHOL ← TC	-1.008	0.264	-3.811	^∗∗∗^	-0.121
CL ← TC	1.000	–	–	^∗∗∗^	0.760
EI ← TC	0.845	0.035	24.109	^∗∗∗^	0.756
IR ← TC	0.953	0.039	24.174	^∗∗∗^	0.763
MR ← EC	1.000	–	–	^∗∗∗^	0.556
UR ← EC	1.560	0.080	19.387	^∗∗∗^	0.842
PM ← EC	1.396	0.071	19.537	^∗∗∗^	0.819
TOBACCO ← TC	-0.058	0.120	-0.485	0.627	-0.014
TOBACCO ← ALCOHOL	0.220	0.012	18.289	^∗∗∗^	0.441
TOBACCO ← EC	-0.153	0.151	-1.011	0.312	-0.029
EC ↔ TC	-0.102	0.011	-8.893	^∗∗∗^	-0.331

*RW, Regression Weights; SRW, Standardized Regression Weights; SE, Estimation of Error; CR, Critical Ratio. TC, Task-involved Climate; EC, Ego-involved Climate; CL, Cooperative Learning; EI, Effort/Improvement; IR, Important Role; PM, Punishment for Mistakes; UR, Unequal Recognition; MR, Member Rivalry. ^∗^p < 0.05; ^∗∗^p < 0.01; ^∗∗∗^p < 0.001.*

Analysis of the influence of indicators on each latent variable showed statistical significance in all cases (*p* < 0.005), with all relationships being positive and direct. In the case of TC, the highest correlation coefficient was obtained with IR (*r* = 0.763), followed by CL (*r* = 0.760) and EI (*r* = 0.756). In the case of EC, the highest correlation was with UR (*r* = 0.842), followed by PM (*r* = 0.819) and MR (*r* = 0.556).

A significant negative and direct association (*p* < 0.005) was observed between TC and alcohol consumption (*r* = -0.121), which was not associated with EC. No association was found between EC or TC and tobacco consumption. A statistically significant (*p* < 0.005) positive and direct association was found between tobacco and alcohol consumption (*r* = 0.441).

**FIGURE 3:**
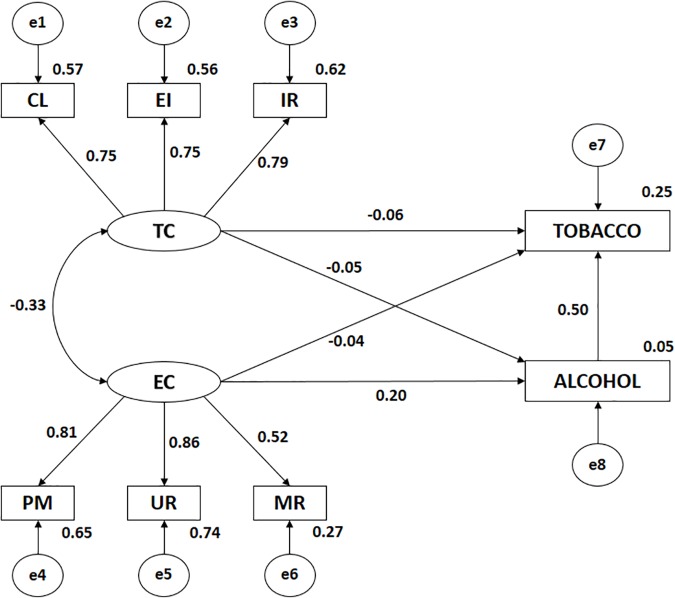
Structural equation model in those practicing another type of physical activity. TC, Climate-Task; CL, Climate-Task Cooperative Learning; EI, Climate-Task Effort/Improvement; IR, Climate-Task Important Role; EC, Climate-Ego; MR, Climate-ego Intra-team Member rivalry; UR, Climate-ego Unequal Recognition; PM, Climate-ego Punishment for Mistakes; TOBACCO, Tobacco Consumption; ALCOHOL, Alcohol Consumption.

Figure 3 and Table 2 exhibit the estimated values of structural model parameters for students who practice another type of physical activity. They should present an adequate magnitude, thee effects should significantly differ from zero and improper estimations should not be obtained, including negative variances. Statistically significant (*p* < 0.005) positive and direct relationships were observed between both TC and EC and their dimensions. There was a significant (*p* < 0.005) negative and indirect relationship between TC and EC (*r* = -0.325).

**Table 2:** Weights and standardized regression weights of those who practice another type of physical activity.

Relationship between variables	RW				SRW
	Estimations	SE	CR	*P*	Estimations
ALCOHOL ← EC	2.327	0.541	4.304	^∗∗∗^	0.196
ALCOHOL ← TC	-0.418	0.387	-1.080	0.280	-0.048
CL ← TC	1.000	–	–	^∗∗∗^	0.753
EI ← TC	0.839	0.049	17.076	^∗∗∗^	0.750
IR ← TC	1.001	0.058	17.323	^∗∗∗^	0.790
MR ← EC	1.000	–	–	^∗∗∗^	0.524
UR ← EC	1.618	0.127	12.771	^∗∗∗^	0.860
PM ← EC	1.469	0.113	12.984	^∗∗∗^	0.809
TOBACCO ← TC	-0.311	0.189	-1.642	0.101	-0.065
TOBACCO ← ALCOHOL	0.274	0.019	14.760	^∗∗∗^	0.497
TOBACCO ← EC	-0.273	0.259	-1.053	0.292	-0.042
EC ↔ TC	-0.099	0.016	-6.092	^∗∗∗^	-0.325

*RW, Regression Weights; SRW, Standardized Regression Weights; SE, Estimation of Error; CR, Critical Ratio. TC, Task-involved Climate; EC, Ego-involved Climate; CL, Cooperative Learning; EI, Effort/Improvement; IR, Important Role; PM, Punishment for Mistakes; UR, Unequal Recognition; MR, Member Rivalry. ^∗^p < 0.05; ^∗∗^p < 0.01; ^∗∗∗^p < 0.001.*

Analysis of the influence of indicators on each latent variable found statistically significant (*p* < 0.005) positive and direct relationships in all cases. In the case of TC, IR showed the highest correlation coefficient (*r* = 0.790), followed by CL (*r* = 0.753) and EI (*r* = 0.750), while EC showed the highest association with UR (*r* = 0.860), followed by PM (*r* = 0.809) and MR (*r* = 0.524), as in the model for physical activity in a natural environment.

A significant (*p* < 0.005) positive and direct association was found between EC and alcohol consumption (*r* = 0.196), which showed no association with TC. As in the model for physical activity in a natural environment, no association was found between EC or TC and tobacco consumption.

A statistically significant (*p* < 0.005) positive and direct relationship was observed between tobacco consumption and alcohol consumption (*r* = 0.497). The relationship was stronger in the case of those practicing activities in a natural environment.

## Discussion

The SEM developed in this study reveals an excellent fit in all evaluation indexes, as a function of the number of participants, and significance was reached. A multigroup analysis of structural equations was conducted to compare the associations between sport-related motivational climate and harmful substance consumption according to the type of physical activity (in natural environments or otherwise). The path model developed achieved excellent fit indexes and represents a valid explicative model to elucidate the relationships between motivational factors and alcohol and tobacco consumption in Spanish secondary school students, in line with various national and international studies (Jaakkola et al., 2016; Lee and Chelladurai, 2016; Stenling et al., 2017; Chacón et al., 2018; Chacón-Cuberos et al., 2018).

Analyzing motivational climate, the proposed structural model revealed a significant inverse relationship between TC and EC among students practicing physical exercise either in a natural environment or in other settings, observing a stronger and more differentiated association among the former. Thus, individuals who practice physical-sports activities in a natural environment perceive a high climate of involvement with the task and a low climate of involvement with the ego, the opposite of the perceptions of those practicing activities in other settings (Navarro-Patón et al., 2018; Ring and Kavussanu, 2018). It appears that students adopt a predominant orientation, either towards the task, rewarding effort and self-improvement, or towards the ego, fomenting rivalry among group members and the pure demonstration of skills (Gano-Overway et al., 2017; Erturan-Ilker et al., 2018). In the case of those practicing sport in a natural environment, this inverse relationship is stronger, explained by the greater group cohesion and the ludic component of physical activities in this environment, promoting cooperation among group members. In contrast, physical activities in other environments place a higher value on the defeat of rivals and the exhibition of their skills (Arslanoglu, 2016; Amaro et al., 2017; Castro-Sánchez et al., 2019).

The most influential category for the TC dimension was IR, while the most influential indicator for EC was UR, especially in the case of students who did not practice physical activity in a natural environment. Harwood et al. (2015), reported that students who assign greater importance in their physical activity practices to social relationships, characteristic of activities developed in a natural environment, show a higher task orientation, prioritizing the ludic component of this type of activity. In activities developed in natural environments, the competitive desire to stand out in the group is attenuated in favor of a greater enjoyment of the activity, focusing on the fun and play it offers, dissociated from the competitive component characteristic of sports (Gerdin and Pringle, 2017).

Adolescents who practice physical activity in nature show a lower tendency to consume harmful substances (alcohol and tobacco) than adolescents who do physical activity in other environments. This association is fulfilled with alcohol, but not with the consumption of tobacco. In terms of the relationship between motivational climate and alcohol consumption, a negative and direct association was observed between TC and alcohol consumption in students practicing sport in a natural environment, while a positive relationship between EC and alcohol consumption was found in students practicing other types of physical activity. Accordingly, physical activity in natural environments is related to lower alcohol consumption, associated with task orientation, while activities in other settings is related to higher alcohol consumption, associated with ego orientation. It is considered that the practice of physical activity in natural environments is a protective factor against the consumption of harmful substances, including alcohol and tobacco (Dodge et al., 2017). This is explained by the generally hedonistic aim of physical activity in a natural environment, geared to enjoyment, contrasting with the performance-oriented objective of other physical-sport activities, leading to possible frustrations when the individual fails, favoring the consumption of alcohol and tobacco, among other harmful behaviors.

The relationship between tobacco and alcohol consumption was positive and direct but was stronger and more differentiated among students practicing physical activity in settings other than nature. This is consistent with the aforementioned proposition that physical-sport activities not developed in natural environments may pose a risk of alcohol consumption due to its higher competitive component (Cronholm et al., 2017). In addition, the state of euphoria generated by the positive results in sports competition causes some young people consume alcohol in order to celebrate the achievements. The natural environment enhances the enjoyment and involvement of adolescents in their activities, hence prioritizing task orientation and favoring lower levels of alcohol consumption (Nooijen et al., 2017) and higher adherence to physical activity during adulthood (Mäkelä et al., 2017).

This investigation permitted analysis and verification of the relationships between motivational factors and harmful substance consumption. However, it was not possible to infer cause-effect relationships due to its cross-sectional design, and longitudinal interventional studies are warranted based on programs of physical activity in a natural environment. It would be interesting for future studies that use the mixed methods study designs would be interesting to address this topic, qualitative research design is needed.

The main conclusion of this study is that physical activity in nature has a ludic component that favors a more self-determined motivation that may be related to lower alcohol consumption. Enjoyment and group cohesion prevail in this environment, whereas there is a greater motivational ego orientation in other settings, which may be related to higher alcohol consumption. The task motivational climate, both in sporting contexts and those linked to the practice of healthy physical activity in nature, constitutes a protective factor against the development of harmful behaviors such as the consumption of harmful substances, the development of sedentary habits or poor diet. Therefore, physical-sport activities in a natural environment are considered important to acquire healthy behaviors associated with adherence to physical activity practice and a lower consumption of harmful substances.

We propose the incorporation of activities in natural environments in school programs with the aim of reducing harmful behaviors and improving the health of adolescents through enjoyment-centered physical activities. In addition, it is necessary to promote task-oriented motivational climates in the adolescent stage, both in the context of the practice of daily physical activity and in competitive sport. This is because it relates to healthier behaviors and greater adhesion to the practice of physical activity that will last over time. To achieve this, teamwork should be encouraged, rewarding the effort to learn and personal improvement. On the contrary, excessive competitiveness must be suppressed, linking sports achievement to progress and not results. In this sense, the playful component of physical activity should be given special importance.

## Data Availability

All datasets generated for this study are included in the manuscript and/or the supplementary files.

## Ethics Statement

The research Ethics Committee of the University of Granada approved this study with code 641/CEIH/2018. Written informed consent was obtained from the parents/legal guardians of all participants.

## Author Contributions

RC-C, FZ-O, JP-T, and MC-S conceived the hypothesis of this study. FZ-O, MC-S, and CC-B participated in data collection. RC-C, MC-S, and CS-L analyzed the data. RC-C, JC-Z, and JP-T wrote the manuscript with significant input from MC-S. All authors contributed to data interpretation of the statistical analysis, and read and approved the final manuscript.

## Conflict of Interest Statement

The authors declare that the research was conducted in the absence of any commercial or financial relationships that could be construed as a potential conflict of interest.
